# Advanced Cardiac Resuscitation Evaluation (ACRE): A randomised single-blind controlled trial of peer-led vs. expert-led advanced resuscitation training

**DOI:** 10.1186/1757-7241-18-3

**Published:** 2010-01-14

**Authors:** Thomas C Hughes, Zoeb Jiwaji, Kamaldeep Lally, Antonia Lloyd-Lavery, Amrit Lota, Andrea Dale, Robert Janas, Christopher JK Bulstrode

**Affiliations:** 1 Emergency Department, John Radcliffe Hospital, Oxford OX3 9DU, UK; 2 University of Oxford Medical School, John Radcliffe Hospital Oxford OX3 9DU, UK

## Abstract

**Background:**

Advanced resuscitation skills training is an important and enjoyable part of medical training, but requires small group instruction to ensure active participation of all students. Increases in student numbers have made this increasingly difficult to achieve.

**Methods:**

A single-blind randomised controlled trial of peer-led vs. expert-led resuscitation training was performed using a group of sixth-year medical students as peer instructors. The expert instructors were a senior and a middle grade doctor, and a nurse who is an Advanced Life Support (ALS) Instructor.

A power calculation showed that the trial would have a greater than 90% chance of rejecting the null hypothesis (that expert-led groups performed 20% better than peer-led groups) if that were the true situation. Secondary outcome measures were the proportion of High Pass grades in each groups and safety incidents.

The peer instructors designed and delivered their own course material. To ensure safety, the peer-led groups used modified defibrillators that could deliver only low-energy shocks.

Blinded assessment was conducted using an Objective Structured Clinical Examination (OSCE). The checklist items were based on International Liaison Committee on Resuscitation (ILCOR) guidelines using Ebel standard-setting methods that emphasised patient and staff safety and clinical effectiveness.

The results were analysed using Exact methods, chi-squared and t-test.

**Results:**

A total of 132 students were randomised: 58 into the expert-led group, 74 into the peer-led group. 57/58 (98%) of students from the expert-led group achieved a Pass compared to 72/74 (97%) from the peer-led group: Exact statistics confirmed that it was very unlikely (p = 0.0001) that the expert-led group was 20% better than the peer-led group.

There were no safety incidents, and High Pass grades were achieved by 64 (49%) of students: 33/58 (57%) from the expert-led group, 31/74 (42%) from the peer-led group. Exact statistics showed that the difference of 15% meant that it was possible that the expert-led teaching was 20% better at generating students with High Passes.

**Conclusions:**

The key elements of advanced cardiac resuscitation can be safely and effectively taught to medical students in small groups by peer-instructors who have undergone basic medical education training.

## Background

Oxford University Medical School runs a traditional medical school curriculum: three years of pre-clinical training followed by three years of clinical training. All students undergo Basic Life Support (BLS) training in their fourth year. In year five, students have an eight-week module of Musculoskeletal and Emergency Medicine. As part of this module the students learn the core skills of advanced resuscitation. Sixth year students complete a full Advanced Life Support (ALS) course.

The students' resuscitation skills are tested in an Objective Structured Clinical Examination (OSCE), performed on week seven of the eight-week course. If students do not pass this OSCE, they may repeat the OSCE that day and may 'pass with rescue'. If the student still does not pass despite this, the OSCE may be re-attempted a few months later.

Increases in student numbers without a corresponding increase in faculty have made teaching advanced resuscitation skills increasingly challenging. It is difficult for one instructor to cope with groups larger than 10 students while continuing to ensure all students acquire of the practical skills of defibrillation. An initial plan to offer only peer-led resuscitation training to all students was not possible due to resource limitations. It was therefore decided to undertake parallel teaching sessions run by experts and peer-instructors, which facilitated a randomised controlled trial.

## Methods

The peer instructors were volunteers recruited from the group of final-year students who had undertaken the two day University of Oxford Special Study Module in Medical Education. The peer instructors devised the format, content and structure of the teaching, but the aims were specified:

By the seventh week of the course all students must be able to:

- assess a collapsed patient for breathing and circulation.

- perform basic cardiopulmonary resuscitation.

- initiate advanced resuscitation according to the ILCOR guidelines.

- defibrillate safely and effectively using a manual defibrillator.

- recognise and treat ventricular arrhythmias associated with cardiac arrest.

These aims were specified because the time was limited (three hours vs. two days for a full Advanced Life Support/Advanced Cardiac Life Support course). Current evidence suggests that amongst patients with a cardiac cause for cardiopulmonary arrest, ventricular arrhythmias treated early with defibrillation are the patient group most likely to recover to full function following successful treatment [1,2].

The practical skills teaching session lasted 90-120 minutes, and followed a 40 minute lecture on the underlying pathophysiology of cardiac arrest and its treatment which was performed by the same senior doctor (TCH) each time. A 45 minute revision session was also provided the day before the OSCE, which was conducted in the same groups, and at this stage a copy of the OSCE scenario was distributed to all groups.

### Safety

The peer instructors were not directly supervised, as this would have confounded the trial. A senior doctor was always within 20 metres of the room when the defibrillators were being used. Rather than use standard defibrillators, two defibrillators were bought and modified to be able to only give a 2 Joule shock, whatever energy was selected. While no level of shock would be completely free of danger, a two Joule shock would be the therapeutic dose recommended in a 500 gram baby. A small shock is necessary to activate the mannequins - Laerdal Mega-Code Kelly (Laerdal Medical Limited, UK).

A further safety measure was the use of clip-on electrodes rather than the hand-held defibrillation paddles. This was consistent with local defibrillation policy. The defibrillators were marked clearly to show that they were not to be used for therapeutic use, and were kept in a locked room away from clinical areas.

### Outcome measures

The primary outcome measure was:

• proportion of first-time Pass grades

Secondary outcome measures were:

• proportion of High Passes grades

• safety issues during the OSCE

• safety incidents during training

### Statistics and Ethics

A power calculation was done before starting the study to ascertain whether sufficient numbers of students would be able to be recruited to detect an important difference between the groups, if one were to exist. This minimum important difference to detect was decided by the group to be 20%. The pass rate from previous groups taking the OSCE was 90-95%. The null hypothesis (which we sought to reject) was therefore that the pass rate from the peer group would be 20% inferior to that of the expert-led group.

The calculation assumed 60 students in each group, examining for non-inferiority of the peer-led group using a one-tailed test with an alpha value of 0.05 (a 5% chance of wrongly accepting the hypothesis), the power of the study was 93%. This means that there was a 93% chance of a finding a 20% difference between the two groups, were one to exist.

Statistical analysis was carried out using StatExact v8.0 (Cytel Inc) and SPSS v.14 (SPSS Inc). Ethical approval for this study was obtained through the University of Oxford Central University Research Ethics Committee.

### Allocation, randomisation and blinding

The average size of groups that rotate between different specialties in the fifth year of training is 28 students. As outlined above, limitations in space, equipment and teaching staff meant that it was not possible to run a direct comparison of similar group sizes, and therefore the decision was made to limit the maximum size of the peer-led groups to eight students. This potentially reduces the validity of the comparison, but is a reflection of the 'convenience sample' available, and it was thought that having to manage a large group would be quite intimidating for peer instructors who had little previous experience of such situations.

Randomisation was achieved using a program written in Filemaker Pro (Filemaker Inc.), which enabled group size restrictions on the peer-led group to be met. Students were allocated to groups and the same groups were used for the revision session prior to the OSCE.

Assessment was blinded: the OSCE assessor (either consultant or middle grade doctor) did not know in which group the student had received their training.

The Advanced Resuscitation Scenario OSCE checklist (see additional file 1) is closely based on the ILCOR (International Liaison Committee on Resuscitation) guidelines [3]. The scenario starts by testing basic resuscitation skills, and progresses to test advanced resuscitation skills including defibrillation. The ventricular arrhythmias used were ventricular fibrillation, ventricular tachycardia and torsades des pointes. The students were not expected to identify the arrhythmia, only whether or not it needed immediate defibrillation. The ability to differentiate arrhythmias needing defibrillation from others was tested in a written exam taken in the same week.

A pass mark was awarded if the student achieved 23/25 marks and did not perform any unsafe actions such as:

• charging the defibrillator/discharging the defibrillator without intending to do so.

• failing to warn others of defibrillation.

• failing to visually check that no one was touching the mannequin.

• touching the mannequin or the surface on which the mannequin was placed.

A high pass mark required 25/25 marks including all four components of sections 24 and 25, with no hesitation.

The peer-led groups were smaller with a median of seven students per group compared to 12 in the expert-led group. This was possible because the peer instructors were not drawn from the Emergency Department staff and therefore there was no cost to the Emergency Department (other than the one-off equipment costs).

## Results

**Figure 1:**
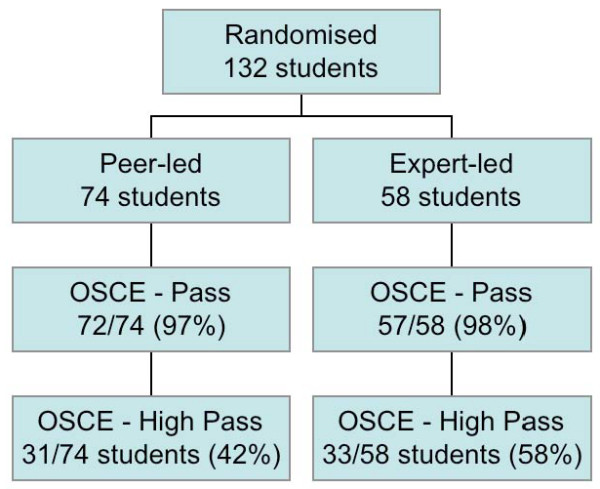
**CONSORT diagram for ACRE study**.

Five rotation groups of students, totalling 132 students were entered into the trial: 74 in the peer-led and 58 in the expert-led group. No students dropped out of the trial. The median rotation group size was 26 (range 25-28), which on average gave one expert-led group of 12 students, and two peer-led groups of seven students (Figure 1: Consort Diagram)

Passes were achieved in the OSCE by 72/74 (97%) in the peer-led group and 57/58 (98%) in the expert-led group. Exact testing confirms that the null hypothesis (that the expert-led group was 20% better than the peer-led group) is rejected (one-sided p < 0.0001). The upper limit of the one-sided 95% confidence limit is 6.5%, well within the 20% limit.

High passes were achieved in the OSCE by 31/74 (42%) in the peer-led group vs. 33/58 (58%) in the expert-led group. Exact testing in this case does not reject the null hypothesis (p = 0.286) as the upper limit of the one-sided 95% confidence limit is 29.6%, and therefore the true difference could exceed the 20% non-inferiority boundary.

No unsafe incidents (see above) related to defibrillation occurred in either the peer-led or expert-led groups. There was one complaint about the teaching quality. This was from a student in one of the expert-led groups wanting to be moved to one of the peer-led groups.

### Expert comparison: Senior Doctor vs. Middle-grade Doctor vs. Nurse Instructor

The subgroup analysis for the High Passes is presented, but due to the small numbers within these group and known hazards of post-hoc statistical analysis, these data should be treated with caution.

All students except one from the nurse-led group passed. For the High Pass grades in:

• the senior doctor/consultant group 6/12 (50%) attained High Passes.

• the middle grade doctor group 11/22 (50%) attained High Passes.

• the nurse group 16/24 (67%) attained High Passes

Analysis of these results did not indicate significant inter-group variation in the proportions awarded Pass (Chi Squared p = 0.472) or High Pass grade (Chi Squared p = 0.213).

## Discussion

### Training need

Defibrillation using automated and non-automated defibrillation is part of the undergraduate acute care curriculum as defined by the ACUTE Delphi survey [4]. Students consistently name the advanced resuscitation training as one of the most enjoyable parts of the Musculoskeletal and Emergency Medicine course.

Peer-led teaching has been successfully incorporated into medical school, and has been used successfully to teach Basic Life Support [5]. Peer-led teaching also appears to have benefits for the peers who act as teachers [6]. The peer-instructors had completed the Medical Education Special Study Module, which included guidance on giving practical instruction and feedback.

The practice of resuscitation is very much along behavioural (stimulus-response) lines, and is assessed using a behavioural assessment tool - the Objective Structured Clinical Examination. Advanced resuscitation, like basic resuscitation is just the chaining of a number of stimulus-response actions.

Advanced resuscitation training involves the use of a defibrillator, and it is likely that this has been the barrier to peer-led training in this area. Using defibrillators that have been modified to ensure no possibility of inadvertent full-energy discharge minimises any risk. The change in defibrillation practice using self-adhesive pads rather than the hand-held paddles beloved of televised emergency drama has also reduced the risk of inadvertent defibrillation of staff.

### Study Design

When this study was first mooted, the question came back "Should medical students be teaching advanced resuscitation to each other at all?" Arguments against allowing students to act as peer instructors centred on either practical issues such as safety and supervision or lack of relevant experience of the peer-instructors. These arguments were particularly vocal from those employed to teach advanced resuscitation.

The first of these objections was answered by using the modified defibrillators and ensuring readily available senior medical supervision. Advanced Life Support courses have used non-medically qualified instructors for many years. The number of resuscitations that these instructors may have led as the main decision-maker will be quite variable. As mentioned above, because the practice of resuscitation is completely protocol-driven, there is no reason that lack of direct experience should be a bar to teaching a practical skill dictated by stimulus-response actions. The theory and evidence base that underpins resuscitation was explained in the 40 minute lecture prior to the practical training.

The peer-led groups were smaller than the expert-led groups. This was a fair comparison as there is no cost barrier to having smaller groups with peer-led instruction, other than one-off equipment costs. However it could be argued that the difference in group size confounded the results.

Judging the quality of educational interventions is difficult. Rather than outcomes, educational studies often use evaluation data from students that is more easily collected and analysed, but much less valid as a judgement of quality. The ultimate test of this intervention would be to measure the number of patients resuscitated by the different groups, but this would not be feasible.

### Randomised controlled trial

Randomised controlled trials are uncommon in medical education [7]. This study was easy to set up as students were already being randomly allocated to the different groups before the study started. The performance of the study therefore did not disadvantage the students in any way.

The most methodologically pure way of assessing the relative worth of any teaching would be the inclusion of a control group who had no teaching at all. However this plan would almost certainly be confounded because peer-led instruction would occur anyway, but would be likely to be inconsistent and would disadvantage those who, for whatever reason, found peer-led instruction difficult to arrange.

### Power

The power of a study is the chance of incorrectly rejecting the null hypothesis - in this case detecting non-inferiority, when this is the reality. In this case the null hypothesis was that the Pass rate from expert-led teaching would be 20% superior to that from peer-led teaching. By performing the calculation at the design stage, we could be sure that we could recruit enough students to be reasonably sure that we could to answer this question. This study had a greater than 90% chance of detecting non-inferiority (in this case by 20%) if in fact this were the case.

The OSCE checksheet appeared sensitive enough to discriminate between candidates as previous groups of students had a Pass rate of approximately 90-95%. It could be argued that the Pass rate, which was higher than expected in both groups, is a result of Hawthorne Effect. However multiple subsequent groups (after the trial had finished) have had maintained this high Pass/High Pass rate, suggesting that this is not the explanation.

### Validity and reliability

#### Content Validity

The content of the Advanced Resuscitation Scenario OSCE checksheet used has been gradually refined over several years of use in assessing medical students and junior doctors. The OSCE has changed as more/better quality evidence becomes is available and is synthesised by ILCOR into clinical protocols and guidelines.

Content validity was ensured by matching the actions to the ILCOR protocols and by incorporating the evidence about situations most likely to have positive outcomes in real-world resuscitation. The OSCE checksheet items were structured with particular emphasis on operator and patient safety and clinical effectiveness.

#### Concurrent Validity/Reliability

Concurrent validity of the OSCE checklist was previously established by measuring the inter-observer variability (kappa) of the form as a whole (pass/fail), and also individual elements within the form, although the form has evolved since that time to reflect the changes made by ILCOR to the resuscitation protocol in 2005.

#### Predictive validity

Predictive validity, while the most important outcome, is very difficult to prove in any study of resuscitation training. This is because the clinical outcomes would be impossible to measure in a way that was ethical, valid and reliable. There is evidence from large-scale trials that training in CPR does make a significant difference to patient survival [8].

#### External Validity

There is nothing in the design or execution of this study that would preclude the technique being used elsewhere. The OSCE checksheet is included as an additional file and may be freely used, copied and distributed, providing the source is acknowledged - see the terms of the Creative Commons licence.

#### Evaluation

Group specific evaluation data is not available, but anonymised evaluation forms were collected for the groups. The evaluations of the ALS teaching by the different groups throughout the year (2007-8) were compared with those from the previous year (2006-7) when peer-led teaching was not used. The evaluation rating was in a range from to one to five, five being the highest.

For the year of the study (2007-8) the mean and SD were 3.92 and 0.41 compared with a 3.83 and 0.59 for the previous year. This difference was not significant (T test for independent samples p = 0.91).

#### Why the difference in High Pass rates?

There are many possible explanations for the experts having a higher High Pass rate: it may be that the experts, and particularly the nurse, who appears responsible for this difference:

• spent more 'hands-on' time rather than theory discussion of ECGs.

• was better at teaching the nuances of 'excellent' rather than just 'very good' resuscitation or

• was a more demanding teacher or

• had more experience of conducting OSCEs and therefore was better able to prepare the students.

Alternatively it may be that the peer-teachers, who were aware that the results of their teaching was being measured, concentrated on making sure that all their students would obtain a Pass grade.

#### Should this course and OSCE replace the ALS course?

The arguments and evidence stated above could be taken to suggest that there is no need for a formal Advanced Life Support (ALS) course, as this short course could equip students with the key knowledge and skills to deal with the emergencies that they are most likely to see and successfully treat. If no definitive course were available, then this would be a sensible arrangement.

However the current arrangement fits well within the model of spiral learning whereby the students learn Basic Life Support in the first clinical year, have this reinforced in the fifth year with the above course that teaches the most important elements of advanced resuscitation, and culminates with a full ALS course in the final year. This structure is particularly important for resuscitation training, as there is a high rate of skills attrition in resuscitation training [9], probably due to the lack of concrete experience to re-enforce the learning on such courses.

## Conclusions

There are caveats about the generalisabilty of this study's results, in that

• the numbers of students in the peer-led groups was limited to 7 for reasons explained above.

• the peer teachers had completed a Special Study Module (SSM) in medical education.

However, with these caveats, this study shows that senior medical student peer-instructors can safely and effectively teach core advanced resuscitation skills to more junior medical students.

## Competing interests

The authors declare that they have no competing interests.

## Authors' contributions

TCH conceived of this study, performed the analysis and prepared the manuscript.

ZJ, KL, AL-L, AL designed and implemented the peer-led teaching programme and contributed to the study design.

AD and RJ participated as expert instructors, contributed to the study design and prepared the figures.

CJKB conceived of the peer-led teaching programme, taught the peer instructors on the Med Ed SSM and arranged equipment necessary for this trial.

All authors read and approved the final manuscript.

## Supplementary Material

Additional file 1 The ACRE resuscitation OSCE Checksheet in Rich Text Format in A4 size. Click here for file

## References

[B1] Norris RM Circumstances of out of hospital cardiac arrest in patients with ischaemic heart disease Heart 2005 91 12 1537 1540 10.1136/hrt.2004.057018 15883135 PMC1769216

[B2] Brindley PG Markland DM Mayers I Kutsogiannis DJ Predictors of survival following in-hospital adult cardiopulmonary resuscitation CMAJ 2002 167 4 343 348 12197686 PMC117846

[B3] 2005 American Heart Association Guidelines for Cardiopulmonary Resuscitation and Emergency Cardiovascular Care Circulation 2005 112 24 Suppl IV1 203 16314375 10.1161/CIRCULATIONAHA.105.166550

[B4] Perkins GD Barrett H Bullock I Gabbott DA Nolan JP Mitchell S Short A Smith CM Smith GB Todd S Bion JF The Acute Care Undergraduate TEaching (ACUTE) Initiative: consensus development of core competencies in acute care for undergraduates in the United Kingdom Intensive Care Med 2005 31 12 1627 1633 10.1007/s00134-005-2837-4 16240145

[B5] Perkins GD Hulme J Bion JF Peer-led resuscitation training for healthcare students: a randomised controlled study Intensive Care Med 2002 28 6 698 700 10.1007/s00134-002-1291-9 12107673

[B6] Buckley S Zamora J Effects of participation in a cross year peer tutoring programme in clinical examination skills on volunteer tutors' skills and attitudes towards teachers and teaching BMC Med Educ 2007 7 20 10.1186/1472-6920-7-20 17598885 PMC1925072

[B7] Torgerson CJ Educational research and randomised trials Med Educ 2002 36 11 1002 1003 10.1046/j.1365-2923.2002.01335.x 12406259

[B8] Stiell IG Wells GA Field BJ Spaite DW De Maio VJ Ward R Munkley DP Lyver MB Luinstra LG Campeau T Improved out-of-hospital cardiac arrest survival through the inexpensive optimization of an existing defibrillation program: OPALS study phase II. Ontario Prehospital Advanced Life Support JAMA 1999 281 13 1175 1181 10.1001/jama.281.13.1175 10199426

[B9] Woollard M Whitfield R Newcombe RG Colquhoun M Vetter N Chamberlain D Optimal refresher training intervals for AED and CPR skills: a randomised controlled trial Resuscitation 2006 71 2 237 247 10.1016/j.resuscitation.2006.04.005 17010497

