# Supplementary material for: Advanced Cardiac Resuscitation Evaluation (ACRE): A randomised single-blind controlled trial of peer-led vs. expert-led advanced resuscitation training
Source: Scand J Trauma Resusc Emerg Med. 2010 Jan 14;18:3. doi: 10.1186/1757-7241-18-3 (PMC2818633; doi:10.1186/1757-7241-18-3)
Supplement: Additional file 1 — The ACRE resuscitation OSCE Checksheet in Rich Text Format in A4 size. [file 1757-7241-18-3-S1.RTF]

Advanced Resuscitation Scenario
"You are on the wards. You are taking a history of chest pain when the patient collapses.
The patient has an i.v. line in situ, and there happens to be a self-inflating bag and mask nearby."
Action	Done	Not done	Prompt	
1.	Safe approach				
2.	Shake and shout				
3.	Call for help / press emergency buzzer				
4.	Opens airway (chin lift/ jaw thrust). Checks for obstruction				
5.	Checks breathing – look, listen and feel for 10 seconds				
6.	Checks for signs of circulation (>5 seconds)				
[optional precordial thump ]	
7.	30 Effective chest compressions – rate >80, <120/min				
8.	Two effective breaths				
"The porter brings defibrillator, but unfortunately cannot stay to help"	
9.	Places pads appropriately on chest				
10.	Turns on defibrillator (without charging it) 				
11.	Diagnoses rhythm as shockable / non-shockable				
12.	Charges defibrillator to 200J (bi) 360J (mono) safely				
13.	Clear warning to others, physically removes oxygen 				
14.	Visual sweep				
15.	Discharges defibrillator safely				
16.	No gap to restart chest compressions				
"It is now two minutes down the track "	
17.	Diagnoses rhythm as shockable / non-shockable				
18.	Charges defibrillator to 200J (bi) 360J (mono) safely				
19.	Clear warning to others, physically removes oxygen.				
20.	Visual sweep				
21.	Discharges defibrillator safely				
22.	No gap to restart chest compressions				
"Let's stop it there……What would you like to do before the next shock?"	
23.	Adrenaline 1mg i.v. with 20 ml flush				
"If this rhythm continues, what further interventions other than more shocks would you consider ?" – at least 2 of:	
Adrenaline - alternate cycles/ every four minutes				
Amiodarone 300mg i.v. with 20 ml flush before fourth shock				
Oxygen +/- Intubation				
4 H's / 4 T's  and name at least five of them				
Total				

Candidate: 			Assessor:				Date:
